# Medical Practitioner’s and Student’s Library

**Published:** 1850-04

**Authors:** 


MEDICAL PRACTITIONER'S AND STUDENTS' LIBRARY.

No. 5 of this series has just been issued by the publishers, Messrs. Lindsay & Blakiston. It is the *American Medical Formulary*, based upon the United States and British Pharmacopœias, by John J. Reese, M. D., Lecturer on Materia Medica and Therapeutics in the Philadelphia Medical Institute. We hope to present our readers with an analysis of its contents in the May number. In the meantine, we can say that we have been favorably impressed with the manner in which the work has been executed, and believe that the flattering opinions expressed in relation to the other volumes of the series will be readily extended to this new aspirant for professional encouragement.

